# Triphasic 3D In Vitro Model of Bone-Tendon-Muscle Interfaces to Study Their Regeneration

**DOI:** 10.3390/cells12020313

**Published:** 2023-01-13

**Authors:** Wendy Balestri, Graham J. Hickman, Robert H. Morris, John A. Hunt, Yvonne Reinwald

**Affiliations:** 1 Department of Engineering, School of Science and Technology, Nottingham Trent University, Nottingham NG11 8NS, UK; 2 Imaging Suite, School of Science & Technology, Nottingham Trent University, Nottingham NG11 8NS, UK; 3 Department of Physics and Mathematics, School of Science and Technology, Nottingham Trent University, Nottingham NG11 8NS, UK; 4 Medical Technologies and Advanced Materials, School of Science and Technology, Nottingham Trent University, Nottingham NG11 8NS, UK; 5 College of Biomedical Engineering, China Medical University, Taichung 40402, Taiwan

**Keywords:** tissue interfaces, indirect 3D printing, 3D cell culture, co-culture, stiffness gradient, regenerative medicine, composite hydrogels

## Abstract

The transition areas between different tissues, known as tissue interfaces, have limited ability to regenerate after damage, which can lead to incomplete healing. Previous studies focussed on single interfaces, most commonly bone-tendon and bone-cartilage interfaces. Herein, we develop a 3D in vitro model to study the regeneration of the bone-tendon-muscle interface. The 3D model was prepared from collagen and agarose, with different concentrations of hydroxyapatite to graduate the tissues from bones to muscles, resulting in a stiffness gradient. This graduated structure was fabricated using indirect 3D printing to provide biologically relevant surface topographies. MG-63, human dermal fibroblasts, and Sket.4U cells were found suitable cell models for bones, tendons, and muscles, respectively. The biphasic and triphasic hydrogels composing the 3D model were shown to be suitable for cell growth. Cells were co-cultured on the 3D model for over 21 days before assessing cell proliferation, metabolic activity, viability, cytotoxicity, tissue-specific markers, and matrix deposition to determine interface formations. The studies were conducted in a newly developed growth chamber that allowed cell communication while the cell culture media was compartmentalised. The 3D model promoted cell viability, tissue-specific marker expression, and new matrix deposition over 21 days, thereby showing promise for the development of new interfaces.

## 1. Introduction

In the musculoskeletal system, tissue interfaces transfer load from soft to hard tissues [1]. Interfaces have limited ability to regenerate after injuries and deterioration, which can prevent the complete healing of injuries and eventually lead to recurrence of the injury after treatment [2]. At present, treatments for repairing damage to musculoskeletal tissues involve surgical interventions, such as grafts and prosthetics to replace or augment the joint. However, these interventions do not aim to repair tissue interfaces [2,3]. The lack of interface regeneration might also lead to graft instability and limited implant-host integration, requiring the replacement of the implant few years after surgery. Tissue engineering approaches could be used to regenerate tissue interfaces to overcome these problems [4,5]. The biology of orthopaedic interfaces is widely known, but the mechanism behind their development is not yet fully understood. 

Bone is a porous structure with pore sizes that increase from 10–50 μm (cortical bone) to 300–600 μm (trabecular bone). The bone matrix is comprised of 60% organic phase, (predominantly type I collagen), and 40% of inorganic phase (calcium, phosphorous, sodium, and magnesium), all of which are organised in crystals and present in the form of hydroxyapatite (HA) (Ca_10_PO_4_OH_2_) [1,6,7,8]. The bone cell population includes osteoblasts, osteoclasts, and osteocytes. 

Tendons are formed mainly of type I and type III collagen fibres, decorin, water, and 0.2% of inorganic phase [9]. The main cell type in tendons is tenocytes. Bones and tendons are connected through the enthesis by mineralised and non-mineralised fibrocartilage [7]. Muscles are composed of bundles of myofibers, specialised multinucleated cells that derive from myoblasts. Each bundle is surrounded by ECM, composed of type I collagen and proteoglycans [10,11]. Tendons and muscles are connected through the myotendinous junction by collagen extensions from the tendon ECM that joins with the muscle fibres [1,7,12]. Currently, the focus is mostly on the regeneration of bone-tendon or bone-cartilage interfaces, with little investigation of the tendon-muscle junction [4]. Numerous studies have investigated individual interfaces, while in the body, multiple interfaces are involved in organ function [4,13] and typical injuries often involve more than one interface. 

When interfaces are studied, it is important to develop gradient scaffolds that gradually change in composition, as well as their physical or chemical properties [14]. Commonly, synthetic polymers like poly (lactic-co-glycolic acid) (PLGA) [5,15,16,17], poly (caprolactone) (PCL) [5,18,19,20], and poly (lactic acid) (PLA) [18,21,22,23] are used for enthesis studies. Bio-glasses [15] or ceramics [16,19,24] can be added to the bone area to resemble the inorganic phase. PCL is also used for MTJ studies [25,26]. The advantage of using synthetic polymers is their easy handling [27]; however, their bioactivity can induce an immune response when implanted [28]. Furthermore, their by-products can be toxic and cell adhesion is not always promoted [29]. Natural polymers do not induce an immune response and can promote cell adhesion and growth, making them a more attractive choice. They can be used to coat the surface of synthetic scaffolds to improve cell adhesion, or they can be used to fabricate entire scaffolds. This, however, comes with reduced mechanical properties so natural polymers are often combined with other materials [27]. Collagen, for example, has been used as a 3D scaffold for a wide range of tissue engineering applications as it is the most abundant protein in the human body, providing support to organs. Thirty different types of collagens are present in the body, and some of these, such as collagen types I, III, V, and XI, have been applied clinically. Type I collagen is one of the most commonly used scaffold materials for numerous different tissues, including bones, tendons, and muscles [30,31,32,33], as it does not induce an immune response [34]. Collagen has suitable mechanical properties, but because covalent crosslinking is not present when it is utilised in tissue engineering, it weakens. To increase its stability, physical, chemical, and natural crosslinkers can be used [35]. Alternatively, other biomaterials like chitosan [36] or agarose [37,38] can be added to improve its mechanical properties. Collagen can also be mixed with calcium phosphates [39] or HA [40,41,42], to mimic mineralized tissues.

Ideally, in the simplest direct approach, synthetic scaffolds should mimic the structure of the ECM. The surface topography, roughness, and elasticity of the substrate [43,44,45] influence cell responses, including cell adhesion, proliferation, migration, morphology, differentiation, and gene expression. Nanopits have been shown to improve the expression of bone-specific markers in human mesenchymal stem cells (hMSCs) [46]. In 2016, Choi et al. increased the stiffness of scaffolds and improved MG-63 proliferation by adding HA to poly lactic-co-glycolic acid (PLGA) scaffolds [47]. The proliferation rate and APL activity were also increased when bovine osteoblasts were seeded on porous PCL scaffolds containing HA [48]. The nanofibrous PCL/gelatin scaffolds designed by Leong et al. promoted human dermal fibroblast proliferation and new matrix deposition over 28 days of culturing [49]. In another study, PCL nanofibrous scaffolds enhanced the metabolic activity of human tenocytes, with alignment along the fibres and matrix production over 14 days [50]. The microgrooved collagen scaffolds developed by Chen et al. promoted the formation of muscle myofibers, alignment of myoblasts, and synthesis of new muscle ECM [51]. Myoblasts differentiated into myotubes with enhanced proliferation, elongation, alignment, and expression of muscle-specific markers when cultured on fibrous PEG scaffolds [52]. Co-culturing in interface studies requires an optimized approach [25,53,54] such as direct co-culture, where different cell types are seeded on the same substrate and cultured in the presence of a medium that promotes the survival of all the cell populations [54]. Cooper et al. co-cultured mouse fibroblasts and osteoblasts on a scaffold for enthesis regeneration. To promote high mineralization in the osteoblasts and low mineralisation in the fibroblasts, the optimal concentration of beta-glycerophosphate, the mineralizing agent, was added to basal medium supplemented with ascorbic acid, foetal bovine serum, and antibiotics/antimycotics [21]. In 2015, Merceron et al. developed a differentiation media by adding horse serum, insulin–transferrin–selenium, ascorbic acid, aprotinin, and antibiotics/antimycotics to basal medium to allow the growth of C2C12 and NIH/3T3 [25]. Since the identification of the most appropriate supplements for different cell populations is time-consuming and costly [54], a device that allows cell-cell communication while each cell type is cultured in its specific medium should be developed. A silicon bioreactor, made of two chambers, separated by a perforated wall, was developed by Harris et al. in 2017. MSCs were seeded on a hydrogel that was placed in the perforated wall between the chambers. Endochondral or ligament differentiation was promoted by adding distinct differentiation media to each of the chambers [55]. To allow articular cartilage repair, Chang et al. optimized a dual-chamber bioreactor that promoted separation between chondrogenic and osteogenic medium using a silicon membrane that also contained the scaffold [56]. In interface studies, the cells’ phenotype and genotype or a combination of both at the interfaces are frequently inadequately investigated or entirely disregarded [23,36,57,58], with studies usually employing culture periods that are too short [25,59,60,61]. 

In this study, we aimed to develop a 3D in vitro model of bone-tendon-muscle to study the regeneration of both interfaces. For this purpose, a collagen/agarose-based composite hydrogel was developed, with a stiffness gradient generated by adding different concentrations of hydroxyapatite (HA). Therefore, the 3D in vitro model was fabricated by indirect 3D printing. A biologically relevant surface topography was designed, consisting of pores for the bone and ridges to mimic the tendons and muscle fibres. Human osteoblast-like cells MG-63, human dermal fibroblasts, and Sket.4U cells were investigated to assess their suitability to be used as bone, tendon, and muscle cell models, respectively. After investigating suitable cell seeding densities for each cell type, cells were cultured individually on the newly developed hydrogels to assess their biocompatibility. The response of cells co-cultured on the 3D in vitro model in a newly developed growth chamber, keeping the cell culture media separated while cells were in communication, was assessed over 21 days.

## 2. Material and Methods

### 2.1. Preparation of Biphasic and Triphasic Composite Hydrogels

Agarose (Fisher Scientific, Loughborough, UK) was mixed with water to a final concentration of 0.75% (*w*/*v*) with different concentrations of hydroxyapatite (HA) nanoparticles (Sigma-Aldrich, Gillingham, UK), namely 0% (*v*/*v*) for the muscle area, 0.2% (*v*/*v*) for the tendon area, and 40% (*v*/*v*) for the bone area. Type I rat tail collagen (Corning, Gillingham, UK) was prepared following the company’s instruction to a final concentration of 3 mg/mL. Therefore, the required volumes of sterile 10x phosphate buffer saline (PBS), sterile 1 N sodium hydroxide (NaOH) (Fisher Scientific, UK) in distilled water (dH_2_O), and agarose/HA solutions were calculated and mixed for each section in individual tubes. To avoid very rapid polymerization of agarose, the solutions were kept in a water bath at 37 °C. Additionally, collagen was kept at 4 °C and added last to the mixture. After polymerization_,_ bone and tendon triphasic gels were crosslinked with 10% (*v*/*v*) oligomeric proanthocyanidins (OPC) in 1x PBS for 60 min at 37 °C and 5% CO_2_. The resultant hydrogels are listed in Table 1.

### 2.2. Determining Young’s Modulus from Compression Testing

To perform compression testing, biphasic and triphasic composite hydrogels were cut with a mould to obtain 2 mm thick discs of approximately 6 mm diameter. Compression tests were performed with the ElectroForce 3200 (TA instruments, New Castle, DE, USA) using a 1N load cells, applying a maximum displacement of 0.2 mm. For each sample, a load (F) vs displacement (δL) curve was plotted. Stress (σ) and strain (ε) were calculated using equations 1 and 2, respectively, and used to plot the stress vs strain curves, thereby facilitating the calculation of the Young’s modulus (E) using Equation (3), where the stress over strain parameter is determined from the linear fit of the plotted data.

(1) $$\sigma =\frac{F}{A}(N/m^{2}),$$

(2) $$\varepsilon =\frac{\delta L}{L},$$

(3) $$E=\frac{\sigma }{\varepsilon }(N/m^{2}),$$

where A is the cross-sectional area of the sample and L is the initial sample thickness. 

### 2.3. Design and Fabrication of the 3D In Vitro Model 

**Figure 1:**
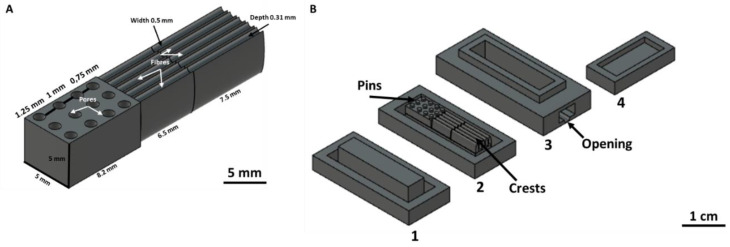
CAD model of the 3D in vitro interface model and vertical mould (**A**) The 3D model was designed with Autodesk fusion 360 with pores (bone section) and ridges (tendon, muscle sections). (**B**) A vertical mould for fabricating the 3D in vitro model was made of a base (1), a support with the negative shape of the surface topography (2), a body to provide the main rectangular shape of the model (3), and a lid to prevent bacterial contamination (4).

The 3D in vitro model was designed with Autodesk Fusion 360 (version 2.0.12392). The model comprises three sections, each with biologically relevant surface topography. A pore gradient was designed for the bone section, and ridged structures to encourage fibres for the tendon and the muscle sections (Figure 1A). The model was fabricated via indirect 3D printing using a mould (Figure 1B), comprising a base to prevent the hydrogel leakage (1), a support with the negative shape of the 3D in vitro model (2), a main body providing a rectangular shape (3), and a lid to prevent bacterial contamination during hydrogel polymerization (4). The mould was fabricated with stereolithographic 3D printing (Form 2, Formlabs, Somerville, MA, USA), in clear resin (base and support, F2-GPCL-04) and tough resin (body and cover, FL-TOT-L05; Formlabs, USA). The parts of the mould were washed and cured after printing to remove uncured resin. The mould was then autoclaved for 20 min at 120 °C. To facilitate the removal of the hydrogel after polymerization, the mould was immersed overnight at 4 °C in sterile 1% (*w*/*v*) Pluronic^®^ F-127. The mould was then assembled, and the triphasic bone gel, the triphasic tendon gel, and biphasic muscle gel were added to the assembled vertical mould and allowed to polymerize in a sterile glass beaker for 60 min at 37 °C and 5% CO_2_. To crosslink the bone and tendon triphasic gels, the mould’s base and support were removed. The body of the mould was placed in a 3D printed sterile bottle with 3 mL of crosslinker for 60 min at 37 °C and 5% CO_2_.

### 2.4. Energy Dispersive Spectroscopy (EDS) with Scanning Electron Microscope (SEM)

To assess the chemical composition, the 3D in vitro model was placed in a freeze-dryer (Christ Alpha 1-2 LDplus, Premier Scientific, Belfast, UK) at −60 °C and 0 mbar, for 8 h. For SEM/EDS analysis, samples were mounted on adhesive carbon tape and coated with 5 nm Au using a Quorum Q150R coater to minimise charge effects. Sample morphology was visualised via secondary electron imaging using a JEOL JSM7100F LV FEGSEM operating at 5.0 kV and a working distance of 10 mm. Qualitative energy dispersive spectroscopy mapping was performed at 20 kV with an Oxford Instruments XMaxn 80 mm^2^ silicon drift detector to determine the elemental distribution, with assignments and data export performed using Oxford Instruments Aztec software (version 3.3 SP1).

### 2.5. Fabrication of the Co-Culture Growth Chamber 

The model was cultured within a growth chamber that comprised a body and a lid. The chamber body was composed of three compartments, one for each tissue, separated by barriers with an opening that allowed the placement of the 3D in vitro model and cell communication. To keep each cell culture medium in its compartment, barriers were also present on the lid. Inlets and outlets were designed to allow the connection of a peristaltic pump for providing a dynamic flow of media to the cells. Leakage and medium isolation tests were performed by adding water stained with different food colours to the compartments. 

To assess the biological inertness of the resin, MG-63 cells were seeded on the bottom of the chamber. After 24 h, haematoxylin and eosin (H&E) staining was performed to verify cell adhesion to the chamber. H&E staining was also performed on a chamber without cells (negative control) and MG-63 cells seeded on a 24-well plate (positive control). 

### 2.6. Response of Cells Cultured on 3D Hydrogels 

MG-63 cells (ATCC^®^, Manassas, VA, USA), human dermal fibroblasts (HDFs) (ATCC^®^, Manassas, VA, USA), and Sket.4U cells (Axiogenesis, Cologne, Germany) were expanded in 2D tissue culture formats. For MG-63 and HDF cells, high-glucose DMEM (Gibco™, Thermo Fisher Scientific, Loughborough, UK) was supplemented with 10% Foetal Bovine Serum (FBS) (Gibco™, Thermo Fisher Scientific, UK), 1% L-glutamine (L-Glu) (Gibco™, Thermo Fisher Scientific, UK), and 1% penicillin/streptomycin (P/S) (Gibco™, Thermo Fisher Scientific, UK). For the Sket.4U cells, a skeletal muscle cell medium (Sigma-Aldrich, UK) was used. 

To identify cell seeding densities where cells reached homeostasis in 3D hydrogels, MG-63, HDF, and Sket.4U cells were seeded with a concentration of 5000 cells/gel (MG-63), 50,000 cells/gel (HDF), or 100,000 cells/gel (Sket.4U) on biphasic gels. Cells were seeded simultaneously on the 3D in vitro model. The model was then placed in the growth chamber and incubated at 37 °C in 5% CO_2_ for 3 h to allow cells to adhere to the model. Then, the chamber was filled with complete medium in the bone section, complete medium in the tendon section, and skeletal muscle medium in the muscle section. 

On days 1, 3, 7, 14, and 21, DNA content, metabolic activity, cell morphology, and expression of tissue-specific markers were assessed with PicoGreen assay (Invitrogen™, Thermo Fisher Scientific, UK), Alamar Blue assay (ThermoScientific™, Thermo Fisher Scientific, UK), histology, and immunocytochemistry, respectively. All assays were performed following the manufacturers’ instructions. 

### 2.7. PicoGreen Assay

At each time point, samples were collected and stored in 0.5 mg/mL Proteinase K (Thermo Fisher Scientific, UK) in 100 mM ammonium acetate (Sigma-Aldrich, UK) at −80 °C until the assay was performed. Then samples were defrosted and incubated overnight at 60 °C to digest the samples. The fluorescence was read with an excitation of 480 nm and emission of 520 nm using a Varioskan Lux 3020 spectrophotometer (Thermo Fisher Scientific, UK). For the 3D interface model and co-culture, n = 24 was determined. 

### 2.8. Alamar Blue Assay

At each time point, the medium was removed, and samples were incubated with the Alamar Blue (Thermo Fisher Scientific, UK) working solution for 3 h at 37 °C and 5% CO_2_. The solutions were then transferred to a 96-well plate, and the absorbance was read spectrophotometrically at 570 nm and 600 nm (Varioskan Lux 3020 spectrophotometer, Thermo Fisher Scientific, UK). For the 3D interface model and co-culture, n = 36 was determined. 

### 2.9. LIVE/DEAD™ Viability Assay

Cell viability was assessed for cells co-cultured on the 3D in vitro interface model with Live/Dead™ (Invitrogen™, UK). The 3D in vitro model was placed in a glass bottom dish 35 mm (Ibidi^®^, Munich, Germany), and the working solution was added to the plate for 30 min at room temperature (RT) in the dark. After incubation, cells were imaged with a confocal microscope (Leica, Wetzlar, Germany). Z-stacks of each section were performed, and 3D projections were created with ImageJ. 

### 2.10. LDH Cytotoxicity Assay

Cell cytotoxicity was assessed for cells co-cultured on the 3D in vitro model with lactate dehydrogenase (LDH) cytotoxicity assay (Invitrogen™, UK). Cell culture medium was collected at different time points and stored at −80 °C until the assay was performed. Absorbance was read at 490 nm and 680 nm (n = 9).

### 2.11. Immunocytochemistry 

The expression of tissue-specific markers was assessed with immunocytochemistry. All primary and secondary antibodies were obtained from Abcam (Cambridge, UK) unless otherwise stated, and 4′,6-diamidino-2-phenylindole (DAPI) was obtained from Sigma-Aldrich (UK). Samples were fixed with 10% formalin (Sigma-Aldrich, UK) for 30 min at RT. Then, cells were permeabilized with 0.1% (*v*/*v*) Triton-x (Alfa Aesar™, UK) in PBS for 5 min. After washing cells three times with dH_2_O, 5% (*w*/*v*) bovine serum albumin (BSA) in 1x PBS was added for 30 min at RT for blocking. Primary antibodies were diluted in 1% (*w*/*v*) BSA to a final concentration of 1:100 for osteonectin and tenomodulin, and 1:500 for α-SMA. Solutions were added for 60 min at RT. Secondary antibodies and DAPI were diluted 1:1000 in 1% (*w*/*v*) BSA. For cells co-cultured on the 3D in vitro model, Donkey Anti-Rabbit IgG H&L (Alexa Fluor^®^ 647) was added to osteonectin, Goat Anti-Rabbit IgG H&L (Alexa Fluor^®^ 488) was added to tenomodulin, and Donkey Anti-Rabbit IgG H&L (Alexa Fluor^®^ 555) was incubated with Alpha-SMA for 60 min at RT in the dark. DAPI was incubated for 15 min at RT in the dark. Cells were imaged with a Leica SP5 confocal microscope (Leica, Germany). Z-stacks of each section were performed, and 3D projections were created with ImageJ. 

### 2.12. Histological Staining

Cell morphology and matrix deposition were assessed with histological stains. For comparison with native orthopaedic interfaces, mouse joints were sectioned and stained. All samples were fixed with 10% formalin (Sigma-Aldrich, UK) for 30 min. Mouse limbs were decalcified for 8 days in 8% HCl (Fisher Scientific, UK) in dH_2_O and 8% formic acid (Alfa Aesar™, UK) in dH_2_O at a ratio of 1:1, at 4 °C. The solution was changed every 2–3 days. 

The dehydration of the 3D in vitro model and mouse limbs was performed with Excelsior™ ES Tissue Processor (Thermo Scientific™, UK). Briefly, samples were incubated in 70% ethanol, 80% ethanol, and 95% ethanol for 60 min each at RT. Then, samples were incubated in xylene for 60 min three times at RT and in paraffin wax at 60 °C for 80 min three times. Embedding in paraffin was performed with HistoStar™ Embedding Workstation (Thermo Scientific™, UK). Samples were sectioned using a microtome (Leica RM2235, Germany), with a thickness of 7 µm. Cells and sections were stained at room temperature using Alizarin red and haematoxylin and eosin stains (H&E). Briefly, 1% (*w*/*v*) alizarin red was dissolved in dH_2_O. The pH was adjusted to 4.1~4.3 with 10% sodium hydroxide (Fisher Scientific, UK). Cells cultured on the 3D in vitro models and mouse limbs were stained for 60 min. For H&E staining, haematoxylin was added for 15 min. Then eosin Y was added for up to 2 min. The slides were dehydrated in 80% and 95% ethanol, each for 1 min, then twice in 100% ethanol for 3 min. Slides were incubated twice in xylene for 10 min before being mounted with DPX mounting medium (Thermo Scientific™, UK). Slides were then imaged at 4x and 40x magnification using the Leica ICC50 W (Leica, Germany) microscope.

### 2.13. Statistical Analysis

Statistical analysis was performed with IBM SPSS^®^. Data were analysed using One-way ANOVA followed by the Tukey post-hoc test with a confidence interval of 99.99% (***), 99% (**), and 95% (*). Graphs were plotted with Microsoft Excel, and statistical significance was added with Inkscape (Inkscape project).

## 3. Results

### 3.1. Fabrication of the 3D In Vitro Model and the Growth Chamber

**Figure 2:**
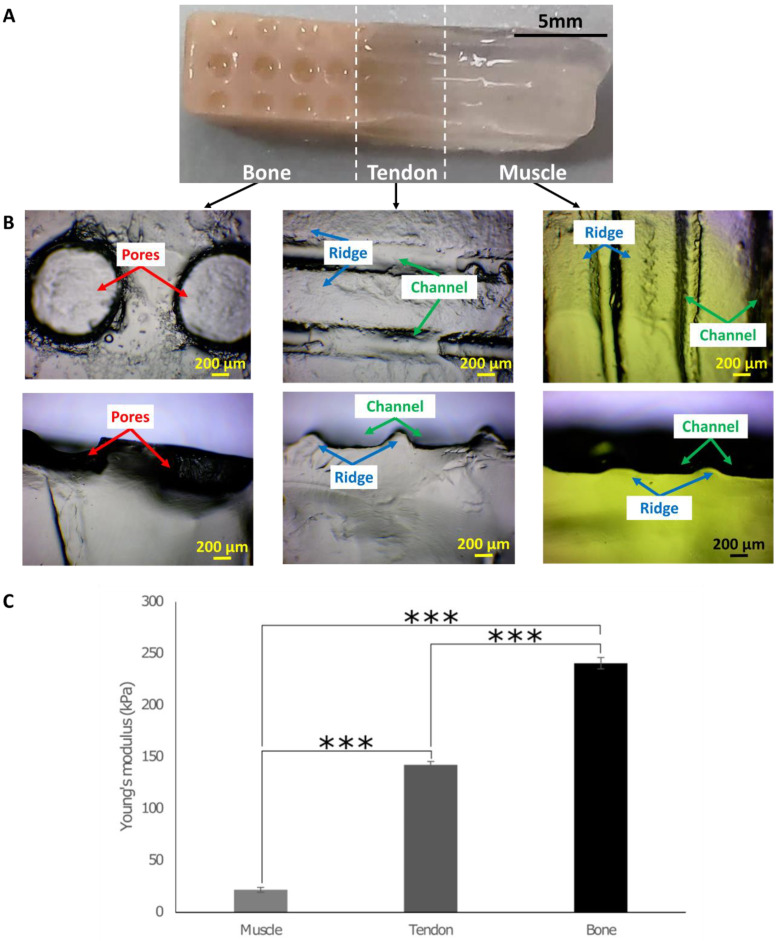
Surface topography and the Young’s moduli of the 3D in vitro model. (**A**) The 3D in vitro model was made of three sections made of 3 mg/mL type I collagen, 0.75% (*w*/*v*) agarose, and a gradient of hydroxyapatite decreasing from the bone to the muscle sections. (**B**) Top and lateral views of the pores on the bone section; the ridges and channels of the tendon and muscle sections. Scale bar = 5 mm and 200 µm. (**C**) The compression test was performed with 1N load of cells. The Young’s modulus was determined from stress vs strain curves. A stiffness gradient was obtained with an increase in the Young’s modulus from the muscle to bone hydrogels. One-way ANOVA and the Tukey post hoc test were performed, *** = *p* < 0.001. Error bars show standard deviation (muscle n = 7; tendon: n = 6; bone: n = 5).

The 3D in vitro interface model was designed with three sections with different surface topographies, namely pores with size gradient for the bone, and ridges and channels mimicking tendon and muscle fibres (Figure 2A). To assess the formation of surface topographies, the mould was tested with different concentrations of agarose, namely 4% (*w*/*v*) (blue), 2% (*w*/*v*) (transparent), and 1% (*w*/*v*) (yellow). Each section was then imaged with a brightfield microscope (Figure 2B). Top and lateral views of the surface topography showed that pores as well as channels and ridges in the tendon section were well defined. In the muscle section, ridges and channels were shorter and not well defined, assumingly due to the lower agarose concentration used. Because type I collagen is the primary component of the bone, tendon, and muscle matrix, it was chosen as the main biomaterial for the 3D model fabrication. However, collagen was not able to form a stable 3D model. In contrast, as stated above, agarose was able to form the 3D model; thus, 3 mg/mL collagen was mixed with 0.75% (*w*/*v*) agarose. This agarose concentration was selected because it was the lowest concentration that provided sufficient support to collagen for the formation of the 3D interface model. To assess the stiffness of the biphasic and triphasic gels, the Young’s moduli were determined using the compression test. The values obtained were ~20 kPa for the muscle biphasic gels, ~140 kPa for the tendon triphasic gels, and ~240 kPa for the bone triphasic gels, resulting in the desired stiffness gradient within the 3D in vitro interface model (Figure 2C).

**Figure 3:**
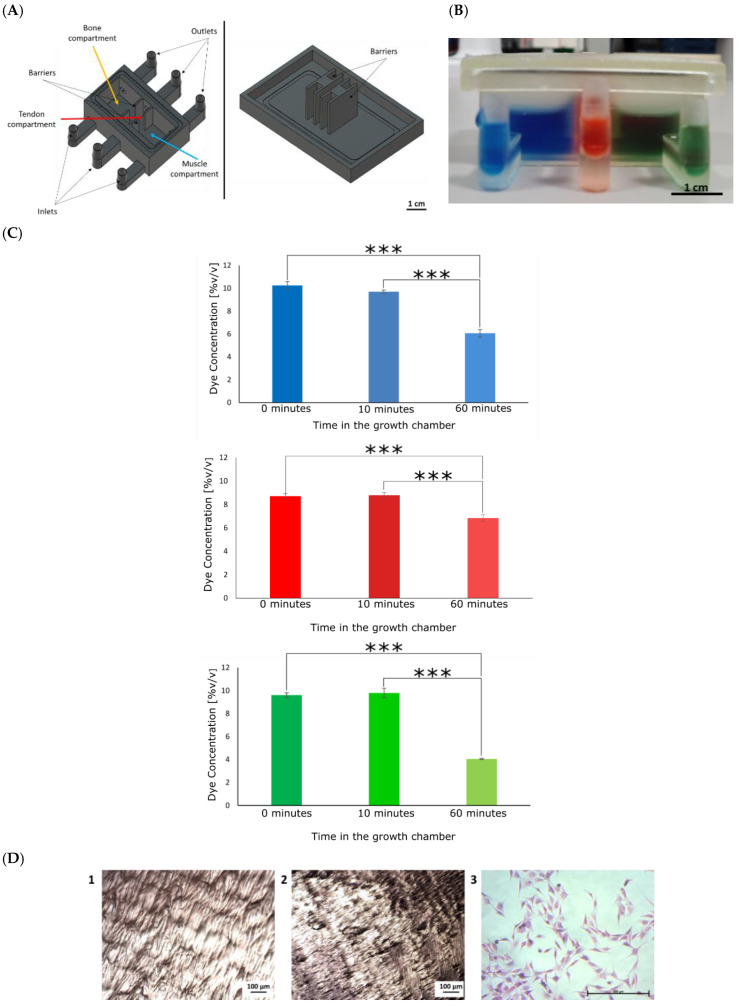
Fabrication and testing of the growth chamber. (**A**) The growth chamber is composed of a body and a lid. The body is divided in three compartments, one each for bones (yellow arrow), tendons (blue arrow), and muscles (red arrow). Both the body and the lid have barriers that keep the media separated. Media inlets and outlets were designed to allow for medium circulation. Scale bar = 1 cm. (**B**). A gel was placed at the bottom of the chamber. Liquids in different colours were added to the chamber. After 10 min, there was no sign of the liquids mixing. Scale bar = 1 cm. (**C**) Concentrations of the blue dye, red dye, and green dye were determined. One-way ANOVA and the Tukey post hoc test were performed, *** = *p* < 0.001. (**D**) MG-63 cells were seeded on the chamber and stained after 24 h. H&E staining of the chamber without cells (1), chamber incubated with cells (2), and MG-63 cells cultured in 2D tissue culture plastic(3). MG-63 cells were imaged at 10× magnification. In both chambers, there were no sign of cells on the bottom. Scale bar = 100 µm (1, 2) and 200 µm (3).

The growth chamber (Figure 3A) was able to keep three coloured liquids separated for 10 min (Figure 3B), indicating the potential to separate tissue-specific media whilst enabling cell communication. The absorbance and the related dye concentrations were measured at 0 min, 10 min, and 60 min after their addition to the chamber (Figure 3C). Although the liquids mixed after 60 min, the amount of mixing was considered acceptable, as in the body, each tissue is not completely isolated from another, and a chemical gradient is normally present. The chamber prevented a complete mixing of the liquids after 24 h. 

To assess the bio-inertness of the resin, MG-63 cells were seeded on the bottom of the chamber. No cells were observed in any of the chambers, and cells were not expected to adhere to the chamber material. The absence of positive staining in both chambers suggested that the chamber is bioinert.

### 3.2. SEM/EDS Analysis to Assess the Composition of Hydrogels

**Figure 4:**
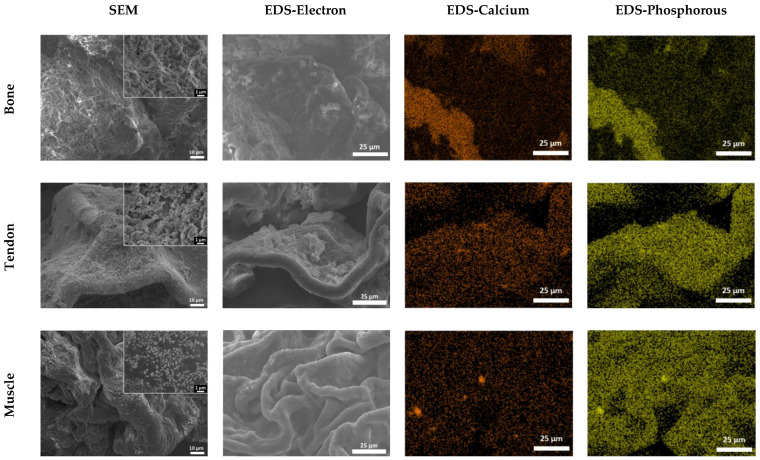
SEM and EDS imaging of the 3D in vitro model. Samples were freeze-dried for 8 h and imaged with SEM. SEM images show the structure of the bone, tendon, and muscle sections at 1000× and 10,000× (inserts) indicated magnifications. Scale bars = 10 µm for 1000× and 1 µm for 10,000×. EDS analysis shows the distribution of calcium and phosphorus within the sections. Images analysed at 1000×, scale bar 25 µm.

**Figure 5:**
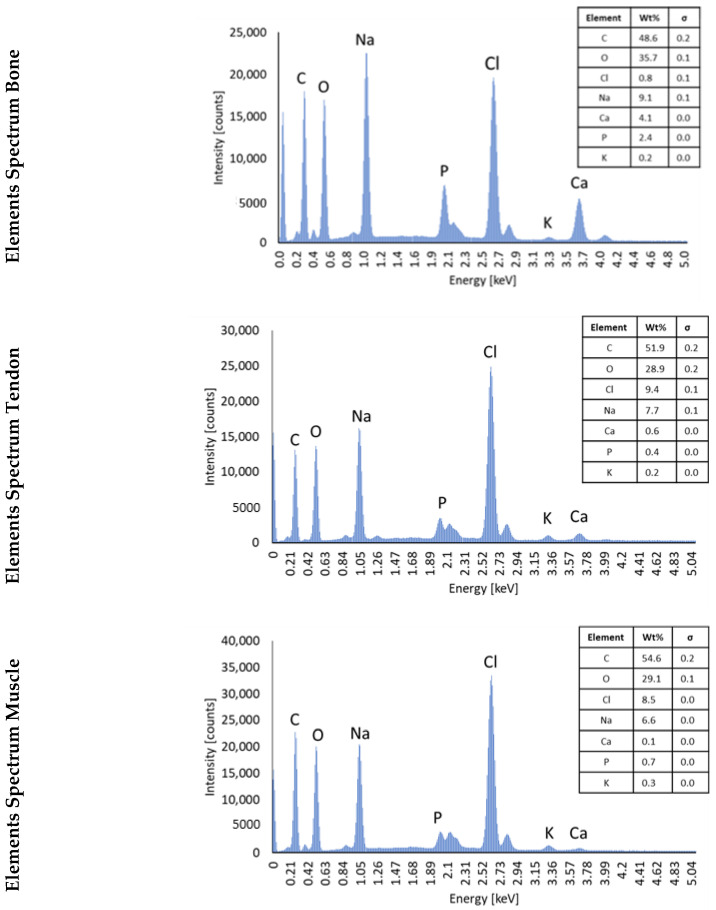
EDS analysis of the 3D in vitro model. EDS spectrum analysis indicates the highest amounts of calcium in the bone and tendon sections. Additionally, together with calcium and phosphorus, there are also high percentages of sodium and chloride.

Figure 4 shows representative SEM images of the bone, tendon, and muscle sections. Secondary electron imaging (EDS electrons) shows the presence of crystals in all the sections. Energy Dispersive Spectroscopy (EDS) suggests that many of the crystals are principally sodium chloride (NaCl). NaCl is a component of PBS that was used to dilute collagen, as described in Section 2.1, explaining why Na and Cl are abundant. A gradient of HA (Ca_10_PO_4_OH_2_), decreasing from the bones to the muscles, was generated within the 3D model. Regions of Ca and P were observed using EDS mapping. Looking at the percentage of Ca and P present in the sections (Figure 5), it can be observed that the abundance of these elements decreases from the bones to the muscles, following the expected trend.

### 3.3. Identification of Cell Homeostasis 

Before cell culture experiments were carried out on the 3D hydrogels and interface model, MG-63, HDF, and Sket.4U cells were assessed in 2D culture for their suitability to act as cell models for the bones, tendons, and muscles, respectively (Supplementary Data, Figures S1 and S2). 

Cells were then seeded on the 3D hydrogels to identify which seeding densities are required for cells to reach homeostasis. This is the point where cells will stop proliferating and start to migrate, leading to the production of new matrix to eventually form the interfaces. 

**Figure 6:**
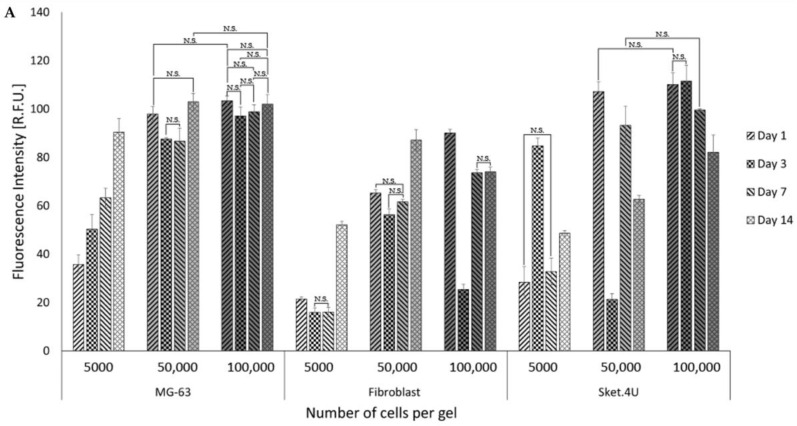

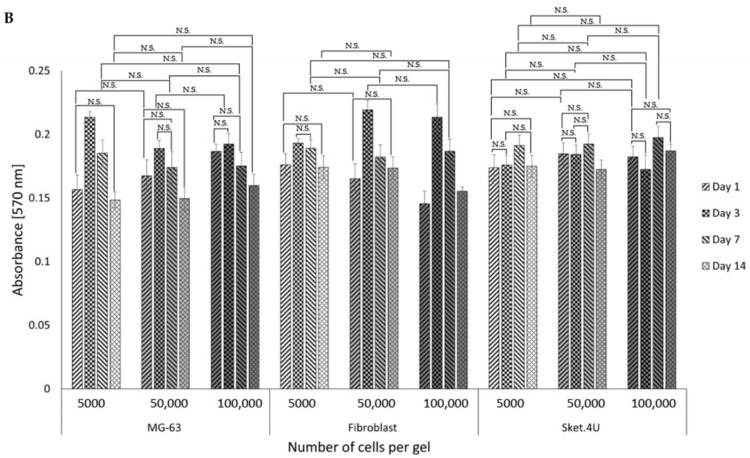
DNA content and metabolic activity assessed for cells grown on biphasic hydrogels. Cells were seeded with a cell density of 5000 cells/gel, 50,000 cells/gel, or 100,000 cells/gels in a 96-well plate to assess homeostasis. On days 1, 3, 7, and 14 after seeding, (**A**) DNA content was quantified using the PicoGreen assay, and the fluorescence intensity was read at an excitation of 480 nm and emission of 520 nm (n = 6); (**B**) the Alamar Blue assay was performed, and the absorbance was read at 570 nm and 600 nm (n = 9). Multi-way ANOVA and the Tukey post hoc test were performed. N.S = not significant; the other values are statistically significant with *p* < 0.05. Error bars show standard deviation. The increase in colour intensity indicates the cell seeding density, while the pattern represents the time point.

Concentrations of 5000 cells/gel, 50,000 cells/gel, and 100,000 cells/gel were seeded on biphasic gels. The DNA content and metabolic activity were assessed with the PicoGreen assay (Figure 6A) and Alamar Blue (Figure 6B), respectively. Cells cultured at 5000 cells/gel did not reach homeostasis, as the DNA content of cells continued to increase with time. The DNA content of 50,000 cells/gel (MG-63 cells) remained stable between days 3 and 7 before increasing to day 14 though not significantly for 100,000 cells/gel. For HDF cells, the DNA content remained similar for all seeding densities until day 3, where cell numbers were lower compared to day 1. For 50,000 cells/gel, the DNA content increased again between days 7 and 14. For 100,000 cells, the DNA content increased by day 7, and then remained stable until day 14. For Sket.4U cells, the DNA content for 50,000 cells/gel decreased by day 3 and did not increase significantly for 100,000 cells/gel per gel. For both cell densities, the DNA content decreased between days 7 and 14. For all cell types and seeding densities, the metabolic activity on day 14 was not statistically different in comparison to that on day 1 (except for 50,000 Sket.4U cells/gel where it decreased). For MG-63 and HDF cells, metabolic activity was higher on day 3 than on day 1. No differences were seen for Sket.4U cells. For 50,000 MG-63 cells/gel and for 100,000 Sket.4U cells/gel, no statistical differences were observed between day 3 and day 7. The metabolic activity decreased insignificantly on day 14 for 100,000 Sket.4U cells/gel. The remaining seeding densities showed significant differences between days 3 and 7, followed by a decrease by day 14. 

Since MG-63 cells showed a higher growth rate than the other cells did when cultured in 2D and on biphasic gels, 50,000 cells/gel was chosen as the most suitable cell seeding density. Because the DNA content for HDF cells increased for both seeding densities and the metabolic activity was not statistically different until day 14, 50,000 cells/gel was selected as the cell seeding density. The DNA content of 50,000 Sket.4U cells/gel and 100,000 cells/gel were similar, indicating that fewer cells were more metabolically active. However, 100,000 cells/gel was chosen as the cell seeding density due to the lack of fibre formation in 2D culture. 

### 3.4. Biocompatibility of Biphasic and Triphasic Gels

Cells were seeded individually on the biphasic and triphasic gels at the previously identified cell seeding densities, namely MG-63 cells were seeded on triphasic gels at 50,000 cells/gel; HDF cells were seeded on triphasic tendon gels at a density of 50,000 cells/gel, and Sket.4U cells were seeded on biphasic muscle gels at a density of 100,000 cells/gel. 

**Figure 7:**
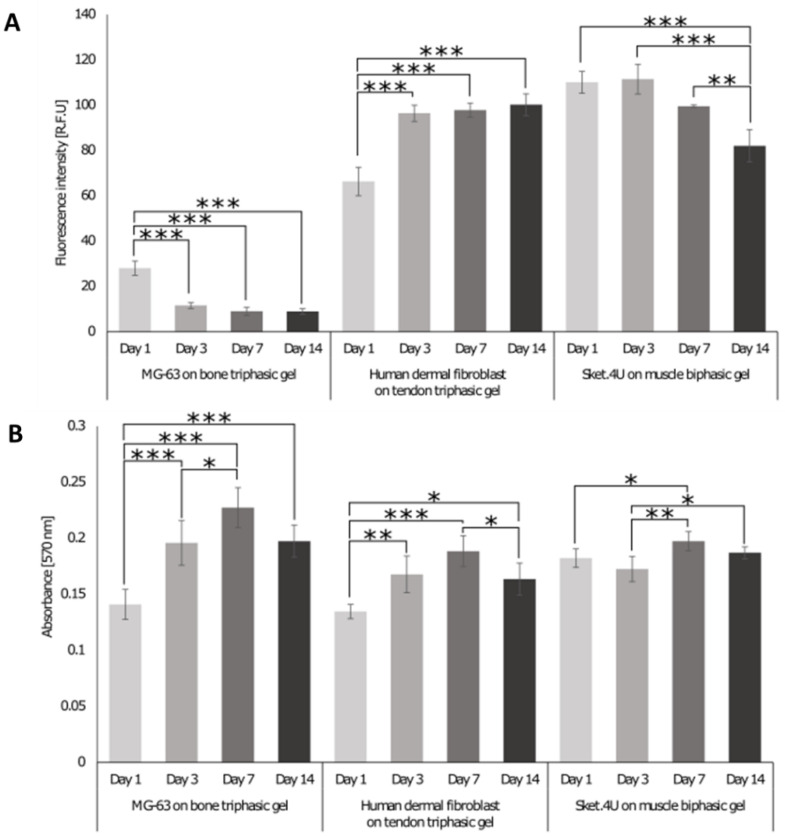
DNA content and metabolic activity assessed for cells seeded on triphasic and biphasic gels. MG-63 and human dermal fibroblast (HDF) cells were seeded with a seeding density of 50,000 cells/gel on the bone and tendon triphasic gels, respectively. Sket.4U cells were seeded on biphasic gels with a seeding density of 100,000 cells/gel. After 1, 3, 7, and 14 days of seeding, (**A**) the DNA content was determined. The fluorescence intensity was read at an excitation of 480 nm and emission of 520 nm. (**B**) The metabolic activity was assessed, and the absorbance was read at 570 nm and 600 nm. The experiment was performed in triplicate, and three readings were obtained per sample (n = 9). One-way ANOVA and the Tukey post hoc test were performed, * = *p* < 0.05, ** = *p* < 0.01, *** = *p* < 0.001. Error bars show standard deviation.

On days 1, 3, 7, and 14, the DNA content (Figure 7A) and metabolic activity (Figure 7B) were assessed. MG-63 cells showed a decrease in the DNA content over time; however, the metabolic activity increased over 7 days and then decreased insignificantly until day 14, indicating that fewer cells were more metabolically active. For HDF cells, the DNA content increased on day 3, and then, it remained stable over 14 days. The metabolic activity increased until day 7, followed by a decrease on day 14. The DNA content of Sket.4U cells remained consistent over 3 days. It then decreased significantly until day 14, while the metabolic activity increased until day 7, but then started to decrease again. 

### 3.5. Biological Response of Cells Seeded on the 3D Interface Model

#### 3.5.1. Comparison of the Growth Chamber versus the Standard Cell Culture Plate

**Figure 8:**
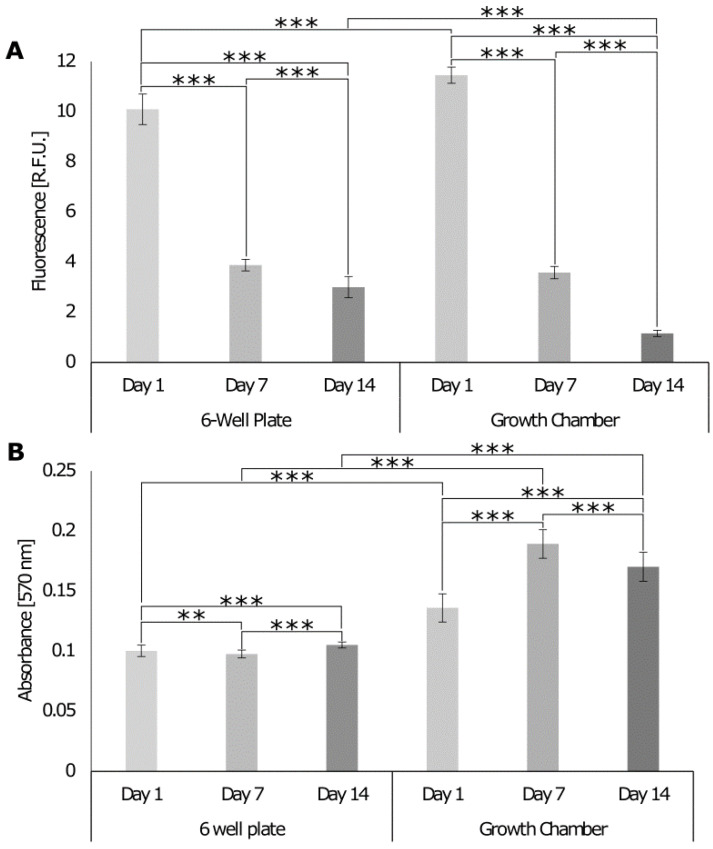
DNA content and metabolic activity of cells seeded on 3D models with topography cultured in a 6-well plate or growth chamber. (**A**) The DNA content was assessed with the PicoGreen assay. The fluorescence intensity was read at an excitation of 480 nm and emission of 520 nm (n = 24). (**B**) The cell metabolic activity was assessed with Alamar Blue. The absorbance was read at 570 nm and 600 nm (n = 36). Two-way ANOVA and the Tukey post hoc test were performed ** = *p* < 0.01, *** = *p* < 0.001. Error bars show standard deviation.

**Figure 9:**
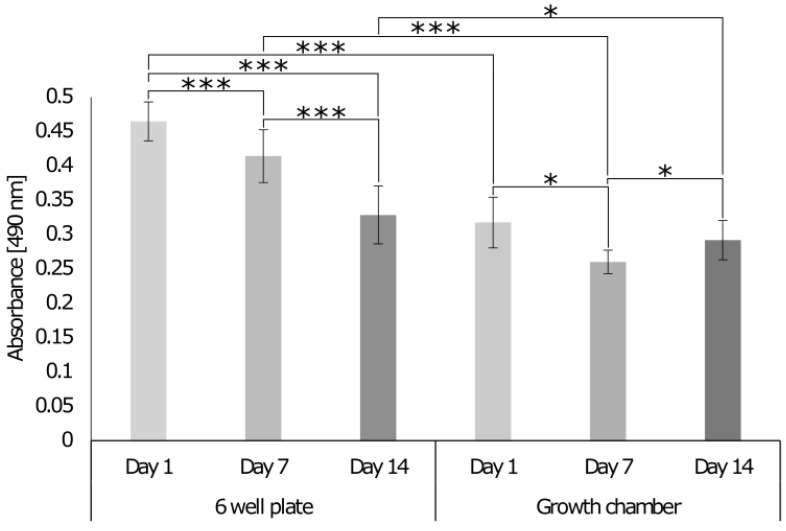
LDH release of cells seeded on a 3D model cultured in a 6-well plate or growth chamber. On days 1, 7, and 14, the release of LDH was detected with the LDH Cytotoxicity Assay. Absorbance was read at 490 nm and 680 nm. Two-way ANOVA and the Tukey post hoc test were performed * = *p* < 0.05, *** = *p* < 0.001. Error bars show standard deviation (n = 9).

To assess whether the chamber allows for cell growth and co-culture, the cell DNA content, metabolic activity, and cytotoxicity were assessed with PicoGreen, Alamar Blue (Figure 8), and lactate dehydrogenase (LDH) cytotoxicity assay (Figure 9), respectively. Analysis was conducted over 14 days, and the results were compared with the response of cells seeded on the 3D model cultured in a standard 6-well plate where the different cell culture media were mixed at a ratio of 4 mL:3 mL:3 mL for MG-63, HDF, and Sket.4U cells, respectively. The DNA content of cells decreased over 14 days for both culture formats (Figure 8A). On day 14, the DNA content of cells was lower for cells cultured in the growth chamber even though the cell metabolic activity increased (Figure 8B). On day 14, the metabolic activity was higher for the cells in the growth chamber than for the cells in the well plate, meaning that the cells were more active even if the DNA content was lower. 

When the cells were cultured in the 6-well plate, a decrease in the level of LDH occurred (Figure 9). In the growth chamber, LDH levels first decreased (day 7) and then increased until day 14. Data normalised to day 1 showed that the variation was lower for the growth chamber, as the values were 0.82 (day 7) and 0.92 (day 14); while for the well plate, LDH levels were 0.89 (day 7) and 0.7 (day 14). However, at the final time point, the LDH release was lower for the cells in the growth chamber than for the cells in the plate, suggesting that the growth chamber was suitable to keep cells alive and active, with reduced levels of cytotoxicity. The higher metabolic activity of cells in the growth chamber compared to the 6-well plate may be due to the separation of the cell culture media. This method was therefore chosen to evaluate the cell response and interface formation over 21 days. 

#### 3.5.2. Biological Response of Cells Cultures on the 3D In Vitro Interface Model 

**Figure 10:**
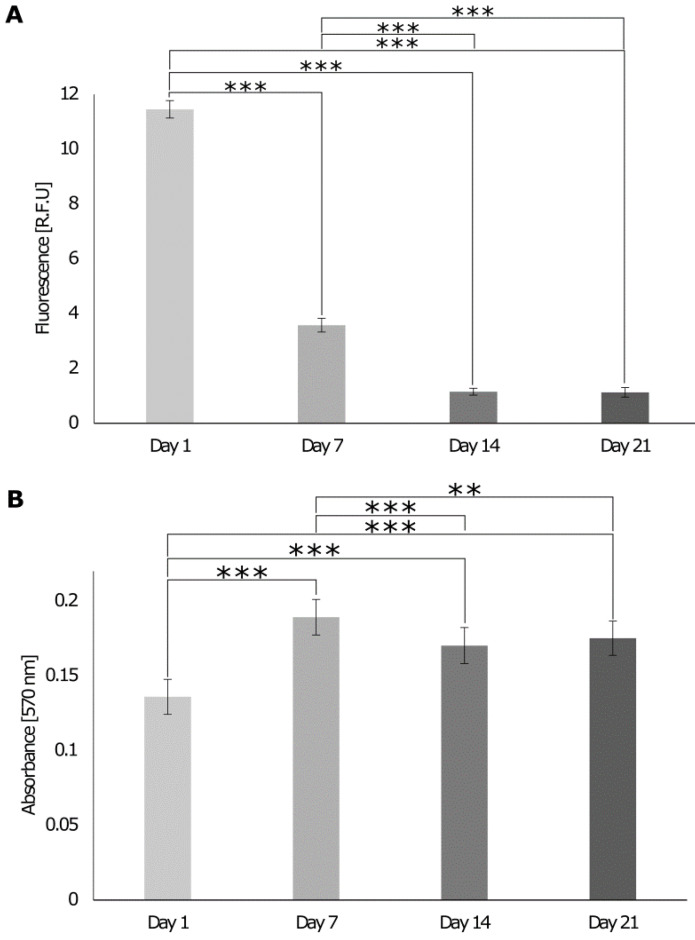
Evaluation of the DNA content and cell metabolic activity of cells seeded on 3D interface model in a growth chamber over 21 days. On days 1, 7, 14, and 21, (**A**) the DNA content was assessed with the PicoGreen assay. The fluorescence intensity was read at an excitation of 480 nm and emission of 520 nm (n = 24). (**B**) The cell metabolic activity was assessed with Alamar Blue. The absorbance was read at 570 nm and 600 nm. One-way ANOVA and the Tukey post hoc test were performed ** = *p* < 0.01, *** = *p* < 0.001. Error bars show standard deviation (n = 36).

**Figure 11:**
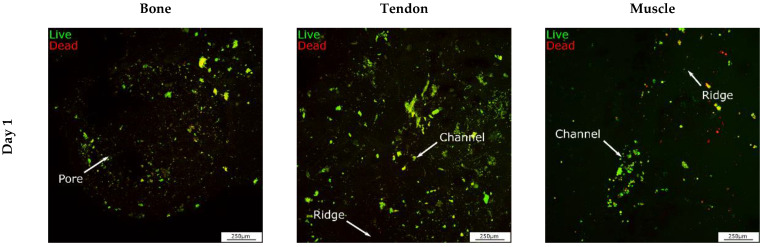

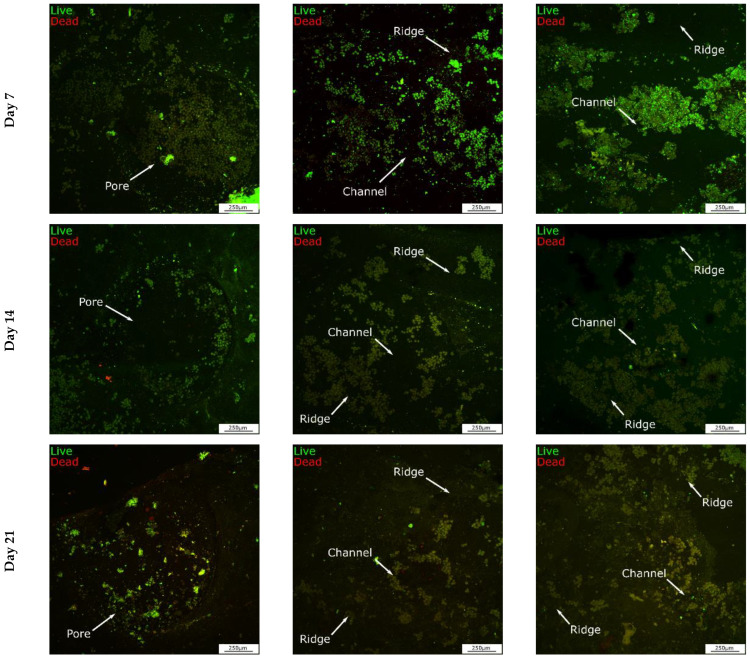
Viability of cells seeded on the 3D interface model cultured in the growth chamber over 21 days. On days 1, 7, 14, and 21, cells were stained with Live/Dead™. Cells were imaged with a confocal microscope with 10× magnification. Live cells are stained green, while dead cells are stained red. Scale bar = 250 μm.

**Figure 12:**
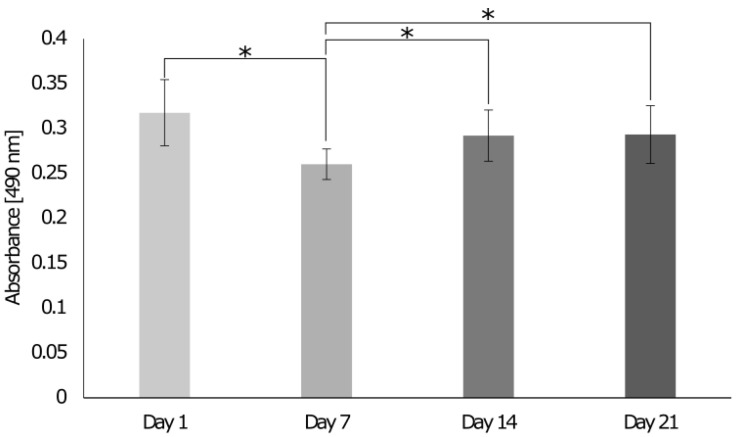
Evaluation of the LDH release of cells seeded on the 3D interface model cultured in the growth chamber over 21 days. On days 1, 7, 14, and 21, LDH release was detected with the LDH Cytotoxicity Assay. The absorbance was read at 490 nm and 680 nm. One-way ANOVA and the Tukey post hoc test were performed *= *p* < 0.05,. Error bars show standard deviation (n = 9).

When cells were cultured on the 3D in vitro model in the growth chamber, the DNA content decreased until day 14 and then stabilized on day 21 (Figure 10A). The metabolic activity of cells increased until day 7 and decreased by day 14, while it stabilized on day 21 (Figure 10B), indicating that cells were still metabolically active and reached homeostasis on day 14. On day 1, various dead cells in the bone and muscle areas were observed (Figure 11), but in general, the number of live cells was higher than the number of dead cells. On day 7, fewer dead cells were detected in all sections and the brightness of living cells increased, suggesting an increase in metabolic activity. Subsequently, the brightness decreased again, and more dead cells were visible on day 14 and day 21. Data were confirmed with the LDH cytotoxicity assay that showed that LDH release decreased on day 7 and increased on day 14 to remain stable on day 21 (Figure 12). 

#### 3.5.3. Assessment of Tissue Interface Development

**Figure 13:**
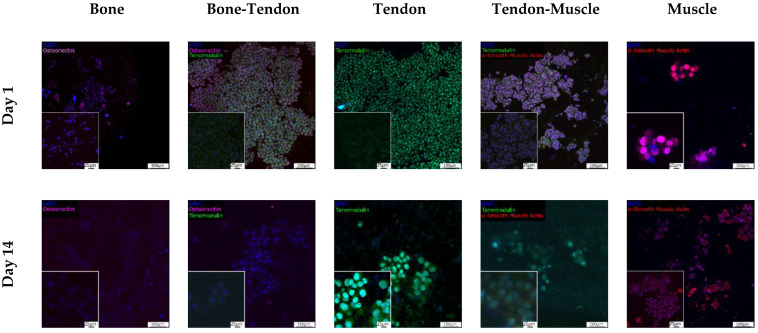
Expression of tissue-specific markers. The expression of tissue-specific markers was assessed with immunocytochemistry. On days 1 and 14, cells were stained with osteonectin (magenta), tenomodulin (green), and αSMA (red). Nuclei were stained with DAPI (blue). Cells were imaged with a confocal microscope. Scale bars are 100 µm for 10× magnification and 25 µm for 40× magnification.

To identify the cell types present in all sections of the 3D in vitro model, the expression of tissue-specific markers was assessed using immunocytochemistry and cells were imaged with a confocal microscope (Figure 13). On day 1, MG-63 cells expressed osteonectin, HDF cells expressed tenomodulin, and Sket.4U cells expressed αSMA. At the bone-tendon interface, both osteonectin and tenomodulin were expressed, while at the tendon-muscle interface, both tenomodulin and αSMA were expressed. By day 14, cell numbers had decreased as indicated by the decrease in the fluorescent signal. However, osteonectin, tenomodulin, and αSMA were still expressed. At the bone-tendon interface, osteonectin was mainly expressed with a low presence of tenomodulin, while at the tendon-muscle interface, a high presence of tenomodulin was observed. 

**Figure 14:**
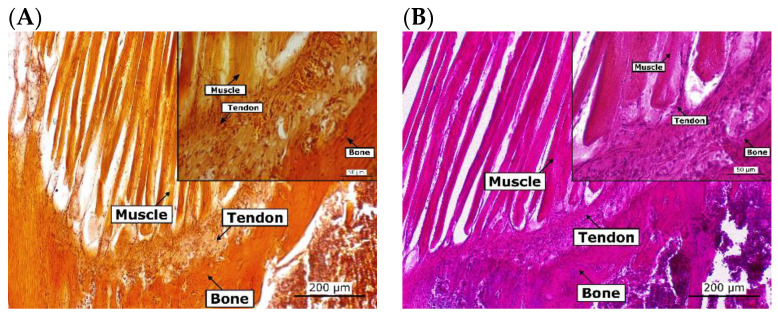
Histological staining of the native tissue. Mouse joints were decalcified for 8 days and paraffin sections of 10 µm were prepared. Paraffin sections were stained with alizarin red (**A**) and haematoxylin and eosin (**B**). Scale bars are 200 µm for 10× magnification and 50 µm for 40× magnification.

**Figure 15:**
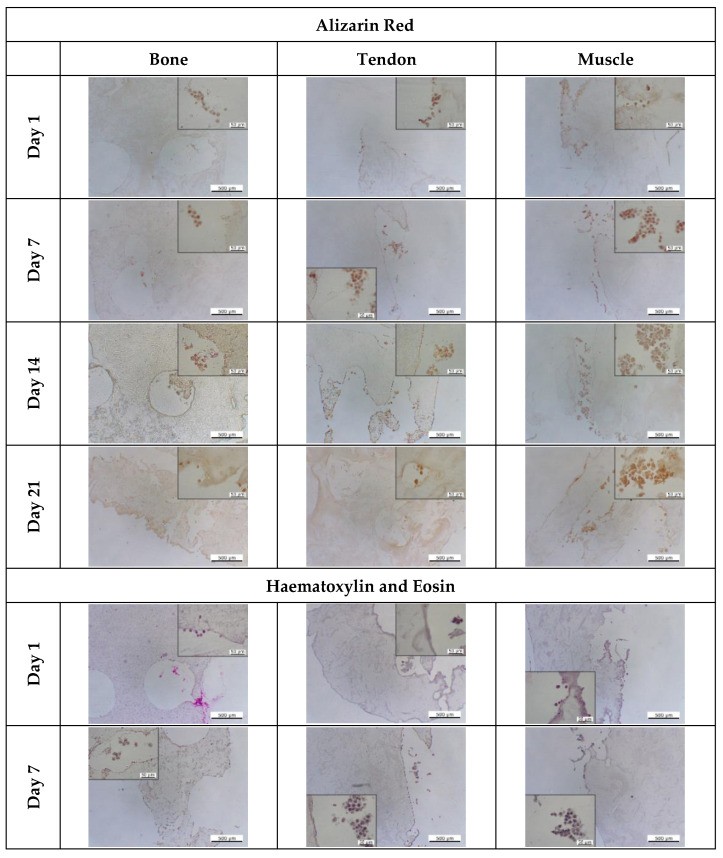

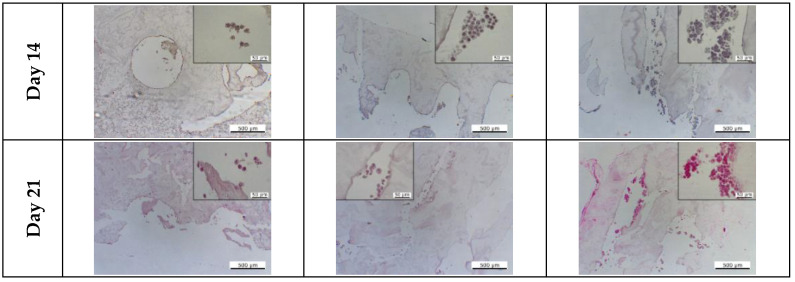
Histological assessment of the 3D interface model for matrix and calcium deposition. On days 1, 7, 14, and 21, the 3D model was sectioned and stained with alizarin red. Alizarin red stains calcium deposits in red/orange. Haematoxylin stains the nuclei purple, and eosin stains the cytoplasm pink. Scale bar are 500 µm for 4× magnification and 50 µm for 40× magnification.

The cell morphology and matrix deposition were assessed with different histology stains (Figure 14 and Figure 15). For comparison with native tissues, mouse joints were also sectioned and stained. Alizarin red staining of the native joint (Figure 14) showed that a higher presence of calcium deposits was observed in the bone and the muscle. Haematoxylin and eosin (H&E) staining showed that cells in the native bone tissue are rounded and randomly organised, while those in the tendon and muscle cells are aligned (Figure 14). Like the native tissues, a higher presence of calcium deposits was observed in the bone and muscle areas of the interface model (day 14) after which all the tissues presented the same colour intensity (Figure 15). The colour intensity for Alizarin Red and H&E staining increased over time for all tissues. Cells started to align in the channels of the tendon and muscle areas; however, fibre formation was not observed. Correspondingly, MG-63 cells did not aggregate to form bone nodules. 

## 4. Discussion 

This study aimed to develop a 3D in vitro interface model by co-culturing MG-63, HDF, and Sket.4U cells in a newly developed growth chamber. When interfaces are studied, the different tissues must be considered. These tissues have different compositions, shapes, and physical properties, which can be mimicked in different layers. The layers can be joined with glues, sutures, or kitting, often resulting in uneven scaffolds [62,63,64]. To obtain scaffolds with a smooth, physiologically relevant transition between the phases, gradients in physical and chemical properties can be developed within the scaffold [14]. Synthetic polymers, like poly (lactic-co-glycolic acid) (PLGA) [5,15,16,17], poly (caprolactone) (PCL) [5,18,19,20], and poly (lactic acid) (PLA) [18,21,22,23] are commonly used because they are easy to handle but can be toxic and do not promote cell adhesion. Natural polymers, such as collagen, a protein present in the bones [1], tendons [65], and muscles [11], are widely used in tissue engineering. Together with the bones [30], tendons [31], and muscles [33], collagen has been also used for tissue engineering of nerves [66,67,68,69], cartilage [70,71,72], and skin [73,74,75]. Collagen was also used in breast cancer studies [76,77]. To enhance the mechanical properties of collagen, it is commonly used with other materials [30,31,32,50,78,79,80,81,82,83,84,85,86,87,88,89,90], such as glycosaminoglycans and calcium phosphates to mimic bones and tendons [82]. Kim et al., in 2013, also used collagen to study the enthesis by mixing collagen with calcium phosphate in different concentrations to mimic the different areas of the enthesis [39]. 

In this study, the 3D in vitro model was composed of three sections with tissue-specific surface topographies (Figure 2A,B). The 3D in vitro interface model was fabricated via indirect 3D printing using a vertical mould with the negative shape of the model. Using indirect 3D printing, it was possible to obtain a 3D in vitro model with a complex shape, made from natural polymers [81]. A stiffness gradient was successfully developed by adding different concentrations of hydroxyapatite, namely 40% (*v*/*v*) for the bone, 0.2% (*v*/*v*) for the tendon, and 0% (*v*/*v*) for the muscle (Figure 2C). The concentrations were chosen, aiming to resemble the inorganic phase of the tissues. In fact, in the native bone, the inorganic phase represents 40% of the total volume [82], while for the native tendon, it is 0.2% [9], and the native muscle extracellular matrix (ECM) does not have inorganic components [11]. 

The Young’s modulus of the biphasic muscle gel was about 20 kPa. In a healthy human body, the muscle’s elastic moduli range from about 26 kPa (quadriceps, male, 22 years old) [83] to about 237 kPa (supraspinatus, male and female, 50 years old) [84]. The Young’s modulus of the triphasic tendon gel was about 140 kPa. The elastic moduli of tendons vary from around 8 kPa (Achilles’ tendon, female, 40 years old) [85] to about 4.5 × 10^5^ kPa (tibialis anterior tendon, male, 22 years old) [86]. Finally, the Young’s modulus of the triphasic bone gel was about 240 kPa. In the body, the range of the Young’s moduli for the bone is between ~3 × 10^4^ kPa (calcaneus, male and female, 23–67 years old) [87] and ~2.6 × 10^7^ kPa (femur, male and female, 53–93 years old) [88]. The Young’s modulus of the triphasic bone hydrogel was not in the range of that for the native bone. To date, the maximum Young’s modulus reached with crosslinked hydrogels is approximately 80 MPa [89]. Considering that the forces native tissues are subjected to are higher in vivo than in vitro [90,91], reaching the Young’s moduli of native tissues might not be essential to enhance cellular responses. Hence, the values obtained in this study were considered appropriate to resemble the interfaces, as there was an increase in stiffness from the muscle to the bone.

MG-63 cells proved to be suitable to model bone as they expressed osteonectin and were organised in aggregates, reflecting bone nodules. HDF cells can be used as a model for tenocytes as they started to align in the same direction and expressed tenomodulin. Sket.4U cells expressed αSMA and aligned along the gels in the same directions, even though no fibre formation was observed. It has been concluded that it might require a higher cell number or a longer culture period to show organization in fibres. Nevertheless, Sket.4U cells were found suitable to mimic skeletal myoblasts. Cell seeding densities that allowed cells to reach homeostasis individually on biphasic gels were determined. Homeostasis is a “self-regulating process by which biological systems maintain stability while adjusting to changing external conditions” [92]. When cells reach homeostasis, they cease proliferation and migration and instead begin differentiation or new matrix production, eventually forming tissue interfaces [93]. We found that 50,000 cells/gel for MG-63 and HDF cells and 100,000 cells/gel for Sket.4U cells allowed cells to reach homeostasis (Figure 6). 

When seeded on the bone triphasic hydrogel, the DNA content of MG-63 cells decreased until day 7, but then remained constant until day 14. The cell metabolic activity increased until day 7 and remained stable until day 14. The DNA content has been related to cell number, because in cells, the overall level of nucleic acids is constant and strongly regulated, even if the levels of DNA or RNA can vary [94,95]. Therefore, a decrease in DNA content is linked to a decrease in cell number. This means that the cells were metabolically active, even if the cell number did not increase. For these reasons, the bone triphasic hydrogel was found appropriate for cell culture. 

HDF cells on the triphasic tendon hydrogel reached homeostasis after 7 days, as indicated by the stable DNA content between day 7 and day 14. Their metabolic activity increased until day 7 and decreased on day 14. Sket.4U cells on the biphasic hydrogels did not show significant changes in DNA content until day 14. The metabolic activity increased on day 7 and remained stable until day 14. Consequently, the tendon triphasic and the muscle biphasic hydrogels were found suitable for cell culture. 

In tissue interface studies, different tissues are investigated simultaneously, requiring advanced co-culture approaches. When different cells are seeded on the same substrate (direct co-culture), it is essential to consider that different cell types may require different cell media, as these might have distinct functions. One of the approaches involves mixing different media in different ratios, but supplements can affect the other cell types [96]. Instead, supplements appropriate for all cell types can be added to a basal medium [21,25]. Nevertheless, medium optimisation can be time-consuming and costly [54]. Otherwise, a device that maintains the different media physically separated while cell-cell contact is allowed can be developed [97]. This method was examined in this study, where a growth chamber was developed for this purpose. The chamber was made of three compartments, one for each tissue, separated by partial walls that avoided the mixing of media, but allowing the insertion of the 3D in vitro model and cell-cell communication (Figure 3). 

On testing the mixing of liquids within the chamber, limited mixing of the liquids occurred after 24 h. The mixing was marginal and acceptable, as in the body, tissues are not completely isolated and gradually change in chemical composition [98]. Clear resin was easy to sterilise and prevented cell adhesion on its own. This should enhance cell adhesion to the 3D in vitro interface model, being the only biologically suitable substrate for cell growth. The chamber was designed to allow the connection to a peristaltic pump to provide a dynamic medium flow to cells, improving the gas and nutrient circulation and enhancing cell proliferation and migration [99,100,101,102,103,104]. Nevertheless, the cell response in dynamic conditions was not assessed in this study. A comparison of the results obtained in dynamic conditions with the ones obtained in this study would be of great interest. Cell culture studies for tissue engineering purposes frequently have too short culture times to accurately imitate tissue regeneration processes in vitro [25,59,60,61]. Studies that are conducted over shorter time frames cannot sufficiently determine whether the method is appropriate since complete tissue repair and regeneration can take weeks to months [105]. Here, cells were cultured for over 21 days. The results showed that cells on the 3D in vitro model reached homeostasis on day 14; however, the number of cells seeded was not high enough to allow the formation of relevant biological structures, such as bone nodules as well as tendon and muscle fibres. The cell DNA content decreased with time, but cells were metabolically active (Figure 10). Alizarin red and H&E staining confirmed that cells deposited new matrix, which could explain the increase in metabolic activity (Figure 15). 

In literature, cell phenotype and genotype at tissue interfaces are not always investigated [23,36,57,58]. Here, the expression of tissue-specific markers was assessed in all the sections of the 3D in vitro model, namely the bone, bone-tendon interface, tendon, tendon-muscle interface, and muscle (Figure 13). It was assumed that cells expressed tissue-specific markers relevant to the section of the in vitro model they were cultured in. However, the expression of osteonectin, tenomodulin, and α-SMA decreased on day 14. Osteonectin was dominantly present at the bone-tendon interface and tenomodulin at the tendon-muscle interface. The low cell population at this time point may be the cause of the decreasing fluorescent signal. Higher cell seeding densities might be required so that cells can adjust to the transition from a standard 2D culture to the 3D in vitro model. Furthermore, even if osteonectin is widely expressed in mineralised tissues, fibroblast also stained positive for this marker [106]. Thus, the cells expressing osteonectin can be both MG-63 and HDF cells. Additionally, it is important to consider that MG-63 is an osteosarcoma cell line. In another study, when MG-63 cells were co-cultured with fibroblasts, they increased their migration towards the direction of the fibroblasts [107]. Thus, the high presence of osteonectin at the interface could be explained by a higher presence of MG-63 cells compared to HDF cells. Similarly, fibroblasts are known to migrate [108], suggesting they may have migrated to the tendon-muscle interface. Furthermore, when seeded in 2D hydrogels, MG-63 cells showed higher metabolic activity than the other cell types did, while Sket.4U cells were less active (Supplementary Data, Figure S1). This can be another reason why osteonectin and tenomodulin were expressed at higher levels at the interfaces. Finally, in this study, cells were seeded simultaneously. However, because these cell types showed different proliferation rates and metabolic activities, seeding them one at a time might allow establishing stable cell populations, thereby providing more time for less proliferative cells to reach homeostasis. Hence, Sket.4U cells should be seeded first, followed by HDF and MG-63 cells. It would be interesting to test the response of the primary cells of the bone, tendon, and muscle to assess whether this 3D in vitro model could be customized with cells from a patient to regenerate tissue interfaces. Moreover, assessing if the construct can promote mesenchymal stem cell differentiation into bone, tendon, and muscle cells could be of great interest.

## 5. Conclusions

A 3D in vitro model of the bone-tendon-muscle interface was developed and validated. It was type I collagen/agarose based, with a gradient in hydroxyapatite, that decreased from the bone to the muscle. MG-63, HDF, and Sket.4U cells were co-cultured over 21 days. Studies were conducted in a growth chamber that allowed cell culture media to remain separated while allowing cell communication. Cells expressed tissue-specific markers and produced new matrix. The 3D in vitro interface model facilitated the increase in cell numbers and metabolic activities and could potentially allow the formation of tissue interfaces. Applications for this 3D in vitro model include studies of disorders, cancer, or ageing on the interface, as well as drug discovery and drug testing. Furthermore, this new 3D in vitro model could be used for the regeneration of the interfaces in patients who have experienced injuries or degeneration.

## Figures and Tables

**Table 1:** Composition of biphasic and triphasic hydrogels.

Type of Hydrogel	Collagen (mg/mL)	Agarose (% *w*/*v*)	Hydroxyapatite (% *v*/*v*)	Crosslinked
Biphasic (muscle)	3	0.75	0	No
Triphasic (tendon)	3	0.75	0.2	Yes
Triphasic (bone)	3	0.75	40	Yes

## Data Availability

The datasets used analysed during this study are available from the corresponding author on reasonable request. The characterisation of cells cultured on 2D standard tissue culture plastic and their expression of tissue specific markers can be found in the Supplementary Data.

## Supplementary Materials

The following supporting information can be downloaded at: https://www.mdpi.com/article/10.3390/cells12020313/s1, Figure S1: Characterization in 2D of MG-63, human dermal fibroblast and Sket.4U. Figure S2: Cell morphology and tissue-specific markers expression.
