# Supplementary material for: Triphasic 3D In Vitro Model of Bone-Tendon-Muscle Interfaces to Study Their Regeneration
Source: Cells. 2023 Jan 13;12(2):313. doi: 10.3390/cells12020313 (PMC9856925; doi:10.3390/cells12020313)
Supplement: Supplementary file 1 [file cells-12-00313-s001.zip › cells-2097059-supplementary.pdf]

1. Method

1.1. Two-dimensional characterisation of tissue-specific cells

MG-63 (ATCC®, USA), human dermal fibroblast (HDF) (ATCC®, USA) and Sket.4U cells (Axiogenesis, DE) were expanded in 2D. For MG-63 and HDF, high-glucose DMEM (Gibco™, Thermo Fisher Scientific, UK) was supplemented with 10% Foetal Bovine Serum (FBS) (Gibco™, Thermo Fisher Scientific, UK), 1% L-glutamine (L-Glu) (Gibco™, Thermo Fisher Scientific, UK) and 1% penicillin/streptomycin (P/S) (Gibco™, Thermo Fisher Scientific, UK). For the Sket.4U a Skeletal muscle cell medium (Sigma-Aldrich, UK) was used.

To assess the cells' suitability as model cell types for bone, tendon, and muscle respectively, each cell type was seeded with a density of 5,000 cells/well in 24-well plates. On days 1, 3, 7 and 14, DNA content, metabolic activity, cell morphology, and expression of tissue-specific markers were assessed with PicoGreen assay (Invitrogen™, Thermo Fisher Scientific, UK), Alamar Blue assay (ThermoScientific™, Thermo Fisher Scientific, UK), histology, and immunocytochemistry respectively. All assays were performed following manufacturer's instruction (sections 2.6 to 2.11) with slight modifications as described below. For Picco Green and Alamar Blue assay n=9 was used.

Immunostaining was performed by staining cells cultured in 2D with Goat Anti-Rabbit IgG H&L (Alexa Fluor® 488) for Osteonectin and tenomodulin; and Donkey Anti-Rabbit IgG H&L (Alexa Fluor® 555) for α-SMA for 60 minutes at room temperature in the dark. Cells were then imaged with a Leica DM IL LED (Leica, DE) fluorescence microscope. Alizarin red stain was performed for 30 minutes, and all histological stains were imaged with Primovert bright-field microscope (Zeiss, DE).

2. Results

2.1. Characterization in 2D culture to assess cell response

MG-63, HDF and Sket.4U were tested in two-dimensional (2D) culture to assess if they are suitable cell models for bone, tendon, and muscle, respectively. The DNA content of all cells increased significantly with time, while MG-63 and HDF grew faster than Sket.4U (Figure S1A). The cell metabolic activity also increased over time, but not significantly for Sket.4U until day 14 (Figure S1B). Alizarin red stains calcium deposits red to orange, while H&E stains the nuclei purple and the cytoplasm pink. On day 14, more calcium deposition was shown in MG-63 and Sket.4U compared to HDF. Both alizarin red and H&E stains showed that MG-63 tended to form cell aggregates, while HDF and Sket.4U aligned on the same direction, as expected (Figure S2). However, at this stage, Sket.4U did not form fibres. MG-63 stained positive for Osteonectin, HDF were positive for tenomodulin and Sket.4U were positive for Alpha-SMA (Figure S2).

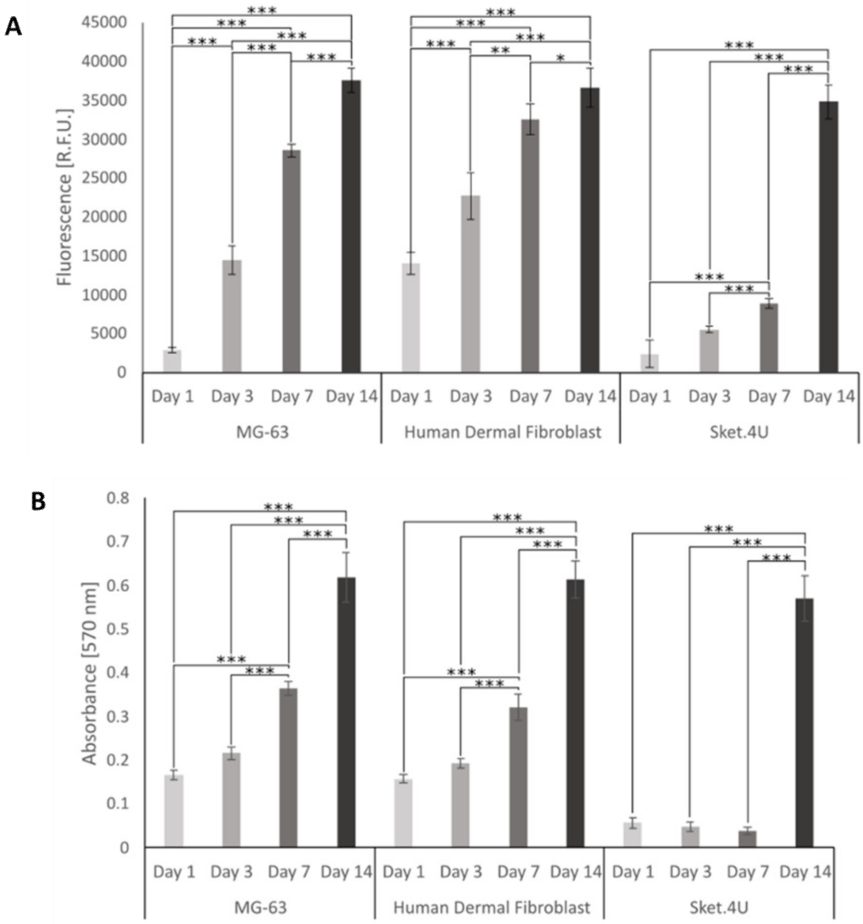

Figure S1: Characterization in 2D of MG-63, human dermal fibroblast and Sket.4U. *Cells were seeded with a cell density of 5,000 cells/well in a 24 well plate. A) After 1,3,7 and 14 days from seeding PicoGreen assay was performed, and fluorescence intensity was read at excitation 480 nm, emission 520 nm. The experiment was performed in triplicate and three readings were done per sample (n = 9). B) After 1,3,7 and 14 days from seeding, Alamar Blue assay was performed, and the absorbance was read at 570 nm and 600 nm. The experiment was performed in triplicate and three readings were done per sample (n=9). One-way ANOVA and Tukey post hoc test were performed, *=p<0.05, **=p<0.01, ***=p<0.001. Error bars show standard deviation.*

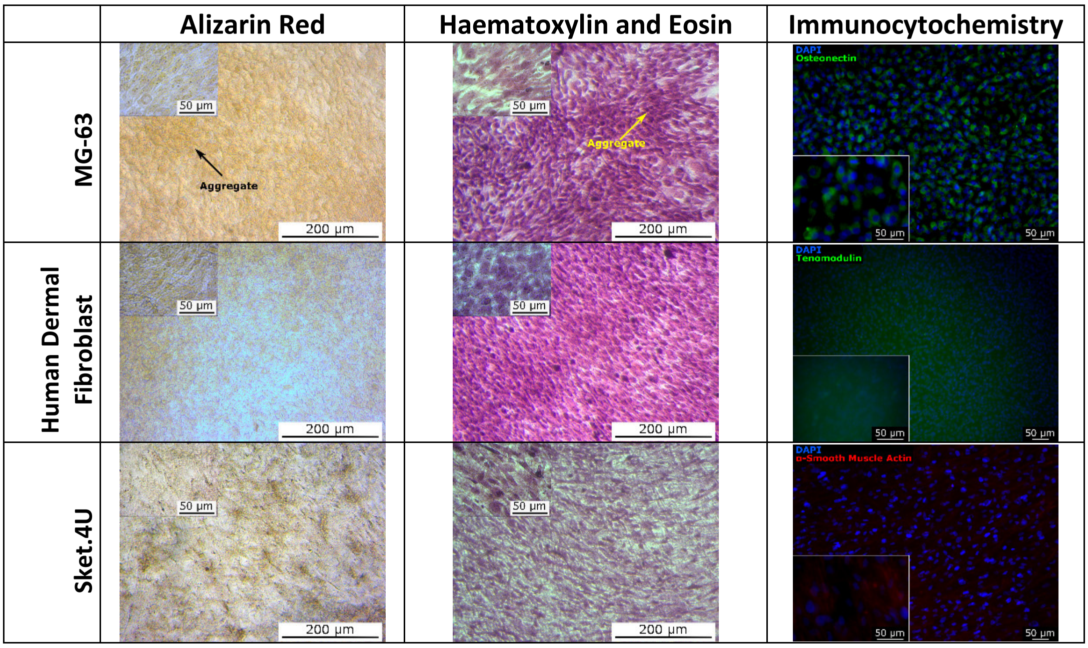

Figure S2: Cell morphology and tissue-specific markers expression. *On day 14 after seeding, cells were fixed with 10% formalin. Cells were then stained with alizarin red for calcium depositions (red) (1), haematoxylin and eosin to visualise cell nuclei (blue/purple) and cytoplasm (pink/orange) (2). Scale bar = 200 µm for 10x and 50 µm for 40x. MG-63 were stained for osteonectin (green), human dermal fibroblast for tenomodulin (green) and Sket.4U for Alpha-smooth muscle actin (red). Nuclei were stained with DAPI (blue) (3). Scale bars= 50 µm.*
